# Role of Specialized Pro-Resolving Mediators in Neuropathic Pain

**DOI:** 10.3389/fphar.2021.717993

**Published:** 2021-08-11

**Authors:** Alessandro Leuti, Marina Fava, Niccolò Pellegrini, Mauro Maccarrone

**Affiliations:** ^1^ Department of Medicine, Campus Bio-Medico University of Rome, Rome, Italy; ^2^ European Center for Brain Research/IRCCS Santa Lucia Foundation, Rome, Italy; ^3^ Faculty of Biosciences and Technology for Food Agriculture and Environment, University of Teramo, Teramo, Italy; ^4^ Department of Biotechnological and Applied Clinical Sciences, University of L'Aquila, L'Aquila, Italy

**Keywords:** resolution of inflammation, chronic inflammation, neuropathic pain, neuroinflammation, resolution of inflammatory pain

## Abstract

Inflammation and neuroinflammation are critical mechanisms in the generation of neuropathic pain that is experienced in several chronic diseases. The aberrant inflammation that triggers this pathophysiologic process can be tracked down to an exacerbated immune response, which establishes a vicious cycle and continuously recruits inflammatory cells by inducing chronic tissue damage. Recently, impairment of the cellular and molecular machinery orchestrated by specialized pro-resolving mediators (SPMs)—i.e., endogenous lipids termed resolvins, protectins, maresins, and lipoxins that confine the inflammatory cascades in space and time during the “resolution of inflammation”–has emerged as a crucial event in the derangement of the inflammatory homeostasis and the onset of chronic inflammation and pain. Indeed, a deviant inflammatory response that is not adequately controlled by the resolution network leads to the overproduction of pro-inflammatory eicosanoids that, opposite to SPMs, lead to neuropathic pain. Interestingly, in the last two decades convincing evidence has demonstrated that SPMs antagonize the *in vivo* activity of pro-inflammatory eicosanoids and, overall, exert potent anti-hyperalgesic effects in a number of pain-associated paradigms of disease, such as arthritis and chemotherapy-induced peripheral neuropathy, as well as in many experimental models of pain like mechanical allodynia, chemical pain, heat hypersensitivity and phase 1 and 2 inflammatory pain. Of note, accumulated evidence supports a synergy between SPMs and other signalling pathways, such as those mediated by transient receptor potential (TRP) channels and those triggered by opioid receptors, suggesting that the cascade of events where inflammation and pain perception take part might be ways more intricated than originally expected. Here, we aim at presenting a state-of-the-art view of SPMs, their metabolism and signalling, in the context of cellular and molecular pathways associated to neuropathic pain.

## Introduction

Pain and chronic neuropathic pain represent a significant social issue, which in Europe affects approximately 20% of the general population (Breivik et al., 2006). This condition can manifest as spontaneous pain, mechanical allodynia–i.e., pain caused by mechanical non-painful stimuli–or hyperalgesia–i.e., increased sensibility to pain (Baron, 2000; Baron, 2006). In general, neuropathic pain arises from a damage of the central or peripheral nervous system occurring during different pathologies, with the sources of this alteration being vastly heterogeneous. Indeed, peripheral neuropathies can arise from a number of localized lesions of the peripheral nerves, from peripheral polyneuropathies (as in diabetes mellitus) or from central lesions that might occur during multiple sclerosis (MS) or spinal cord injury (SCI), as reviewed in Baron (2006). Also enhanced sensitivity of peripheral somatosensory routes is a frequent cause of exacerbated pain, and is elicited by the presence of pro-inflammatory molecules like prostaglandins that are synthesized during acute and chronic inflammation (Jang et al., 2020), as well as by the aberrant activation of the immune response. The pathophysiological elements that trigger this deviant inflammatory response are not yet completely understood, though they have been often linked to persistent inflammation and neuroinflammation (Leuti et al., 2020), which act as a crucial element of neuropathic and inflammatory pain (Kidd and Urban, 2001; Baron, 2006). Inflammation represents a defensive cellular and molecular network, which evolved to avoid the invasion of microbial pathogens that might compromise tissue homeostasis. This process is designed—at least in principle—as a self-resolving mechanism, which is supposed to be confined in space, time and magnitude to avoid tissue damage that abnormal production of pro-inflammatory mediators (e.g., cytokines, eicosanoids) and immune activation would inevitably cause. However, deranged inflammation can lead to a sustained immune response that acts in a vicious cycle, leading to tissue damage, fibrosis, loss of tissue function, and notably to persistent pain by both triggering chronic damage to neural tissues and producing inflammatory mediators that trigger peripheral hypersensitization.

Transition from acute to chronic inflammation has recently emerged as the result of impairment or lack of efficiency of a process termed “resolution of inflammation”, during which a novel class of bioactive lipids called “specialized pro-resolving mediators” (SPMs) would normally coordinate the regression of the immune response by antagonizing both the production and the action of inflammatory mediators that continuously fuel aberrant phlogosis (Serhan and Levy, 2018). Consequently, inefficient resolution caused by insufficient production of SPMs, or lack of response to their homeostatic action, has been shown in a number of independent studies to be associated to chronic pathologies (Leuti et al., 2020). In this context, SPMs work as potent anti-inflammatory/pro-resolving agents which are increasingly emerging as promising targets in the quest for pharmacological strategies able to counteract neuropathic pain during neuroinflammatory diseases.

## Specialized Pro-Resolving Mediators and Their Metabolism

**FIGURE 1:**
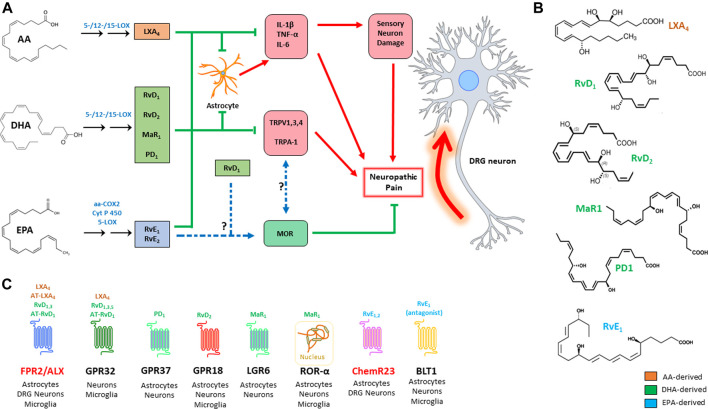
Action of the main SPMs on neuropathic pain **(A)**: SPMs modulate the genesis of neuropathic pain by acting on three main targets: i) inhibition of pro-inflammatory signals; ii) inhibition of TRP channels; and iii) modulation of opioid signalling. Chemical structure of the main SPMs involved in neuropathic pain **(B)**. SPM receptors, with their respective ligands and distribution; receptors that are highlighted in red are those that have been more directly investigated in pain studies **(C)**. AA: arachidonic acid; aa-COX-2: aspirin-acetylated cyclooxygenase 2; ALX/FPR2: formyl peptide receptor 2; AT: aspirin triggered; BLT1: leukotriene B4 receptor 1; ChemR23: chemerin receptor 23; COX: cyclooxygenase; CYP450: cytochrome P450; DHA: docosahexaenoic acid; EPA: eicosapentaenoic acid; GPR: G-protein-coupled receptor; IL: interleukin; LGR6: leucine-rich containin G-protein coupled receptor 6; LOX: lipoxygenase; LX: lipoxin; MaR: maresin; MOR: μ-opioid receptor; PD: protectin; Rv: resolvin; TNF: tumor necrosis factor; TRPV1: transient receptor potential cation channel subfamily V member 1; TRPA: transient receptor potential cation channel subfamily A; ROR: RAR-related orphan receptor.

All SPMs are produced from essential polyunsaturated fatty acids (PUFAs) like arachidonic, docosahexaenoic and eicosapentaenoic acid (AA, DHA, and EPA, respectively), that are introduced in the organism through the diet. These are esterified to the membrane phospholipids, before being released through the action of phospholipase A_2_ (PLA_2_). These precursors are converted into the final products by the concerted action of different lipoxygenase (LOX) isozymes, namely 5-, 12- and 15-LOX, expressed by granulocytes, macrophages, platelets, and endothelial cells. The synthesis of these compounds is triggered by acute inflammation, so that they can antagonize the phlogistic signals by counteracting granulocyte chemotaxis and activation of both innate and adaptive immune cells. To this aim AA, DHA and EPA derivatives trigger differentiation of tolerogenic macrophage phenotypes, by inducing clearance of dead cells and tissue debris and, overall, orchestrating tissue regeneration, and return to normal tissue homeostasis (Chiurchiù et al., 2016; Serhan and Levy, 2018; Leuti et al., 2020). The main metabolic pathways that lead to the synthesis of SPMs, along with their known target receptors, are illustrated in Figure 1.

### AA-Derived SPMs

Lipoxin A_4_ (LXA_4_) and LXB_4_ are the only two known members of this group, and are synthesized by the concerted action of granulocytes, platelets, endothelial cells, and macrophages from phospholipid-derived AA. Their production involves two biosynthetic pathways: the first is in common with that of leukotrienes, because leukotriene A_4_ (LTA_4_) is converted into LXA_4_ and LXB_4_ in a 12-LOX-dependent manner (Serhan et al., 1986). The second route involves 15-LOX-dependent hydroperoxidation of AA, and its 5-LOX-driven conversion into an epoxide, which is eventually hydrolyzed to yield the final LXs (Romano et al., 2015). Acetylation of cyclooxygenase-2 by aspirine (aa-COX-2) gives rise to a series of LX epimers, called aspirin-triggered (AT) lipoxins AT-LXA_4_ and AT-LXB_4_ (Clària and Serhan, 1995). Formyl peptide receptor 2 (FPR2), also known as ALX, and GPR32 mediate the effects of LXA_4_ and AT-LXA_4_, and remain as yet the only receptors known to bind LXs (Chiang and Serhan, 2017).

### DHA-Derived SPMs

DHA-derived SPMs represent the largest group of SPMs, and include three main classes: D-series resolvins 1–6 (RvD_1-6_), maresins 1 and 2 (MaR_1_ and MaR_2_), and (neuro)protectins 1 and X (PD_1_ and PD_X_). These SPMs are produced by LOX-catalyzed differential insertion of oxygen in the carbon backbone of DHA, to yield one of two hydroperoxyls (Hp): i) 15-LOX yields a 17(*S*)-hydroperoxyl derivative (17(*S*)-HpDHA), which is then converted into different RvDs or into PD_1_ by 5-LOX; ii) alternatively, 12-LOX yields 14(*S*)-HpDHA, which can be converted into its corresponding 13(*S*)-14(*S*) epoxide, and then is hydrolyzed into the MaR series. Interestingly, DHA can also be converted into 17(*R*)-HpDHA by aa-COX2, and the latter compound is converted by 5-LOX into a group of epimeric substances called aspirin-triggered (AT)-RvDs (Serhan and Levy, 2018).

Despite the considerable number of molecules that have been characterized in this group—which also includes the corresponding cysteinyl derivatives of Rvs, protectins and MaRs—only a handful of receptors have been discovered so far: RvD1, RvD3, and RvD5 engage GPR32, while RvD1 is also able to bind (much alike LXA_4_) FPR2/ALX (Figure 1C). In addition, RvD_2_ engages GPR18, while PD_1_ and MaR_1_ have only recently been characterized as agonists of GPR37 and LGR6, respectively (Serhan and Levy, 2018). Of note, MaR_1_ is also able to bind the intracellular retinoic acid-related orphan receptor α (RORα), as recently reported (Han et al., 2019).

### EPA-Derived SPMs

EPA derived E-series resolvins (i.e., RvE_1-3_) are synthesized in a rather peculiar way, if compared to the other SPMs, because the synthesis of their main common precursor, 18 (*R*)-HpEPE, is catalyzed by aa-COX2 in vascular endothelial cells, or alternatively by cytochrome P450 (CYP450). Then, 18 (*R*)-HpEPE can be converted by 12-LOX and 15-LOX into RvE_3_, while 5-LOX converts it into RvE_1_ and RvE_2_. E-series resolvins are rather unique in the way they engage their target receptors during resolution of inflammation: in particular, RvE1 is the only known pro-resolving lipid that acts both as an agonist to its own receptor—i.e., Chemerin Receptor 23 (ChemR23)—and as an antagonist to the leukotriene B_4_ receptor 1 (BLT1) (Serhan and Levy, 2018). Instead, all the other SPMs seem to exclusively activate pro-resolving receptors, often being able to act on different targets. Finally, RvE_2_ acts as a partial agonist to ChemR23 (Oh et al., 2012).

## SPMs in Pain

The antinociceptive activity of SPMs has been mostly correlated to their ability to counteract the inflammatory surge that is unleashed during inflammatory response, which in turn causes both hyperactivation of pain neural routes and inflammatory damage leading to chronic neuropathic pain. However, in the past 2 decades SPMs have been consistently proven to act on pain perception via a cross-talk with other signal transduction systems that directly control nociception at the neural level, such as those driven by opioid receptors and transient receptor potential (TRP) channels. In particular, TRP receptors are cation-permeable membrane channels which are gated by physical (e.g., hot or cold) or chemical (e.g., endogenous lipids, or molecules found in foods and spices) stimuli; upon their activation, they drive the depolarization of the dorsal root ganglia (DRG) sensitive neurons by causing Ca^2+^ and Na^+^ influx (Jardín et al., 2017). Relevant to this work, SPMs represent the first endogenous inhibitors of TRPs documented so far (Park et al., 2011b; Lim et al., 2015). The main effects of AA-, DHA- and EPA-derived SPMs on neuropathic pain models are summarized in Table 1.

**TABLE 1:** Main effects of AA-, DHA-, and EPA-derived SPMs on neuropathic pain. AA: arachidonic acid; aa-COX-2: aspirin-acetylated cyclooxygenase 2; ALX/FPR2: formyl peptide receptor 2; AT: aspirin triggered; BDNF: brain-derived neurotrophic factor; BLT1: leukotriene B4 receptor 1; CFA: complete Freund’s adjuvant; ChemR23: chemerin receptor 23; COX: cyclooxygenase; CYP450: cytochrome P450; DHA: docosahexaenoic acid; DM: diabetes mellitus; DRG: dorsal root ganglia; EPA: eicosapentaenoic acid; EPSC: excitatory postsynaptic current; GPR: G-protein-coupled receptor; LGR6: leucine-rich containin G-protein coupled receptor 6; IL: interleukin; LOX: lipoxygenase; LX: lipoxin; MIA: monoiodoacetate; MaR: maresin; MOR: μ-opioid receptor; NDMA: *N*-methyl D-aspartate; OA: osteoarthritis; PD: protectin; Rv: resolvin; SCI: spinal cord injury; TRPV1: transient receptor potential cation channel subfamily V member 1; TNF: tumor necrosis factor; TRPA: transient receptor potential cation channel subfamily A; ROR: RAR-related orphan receptor.

AA-derived SPMs	Effects	Disease model	Reference
LXA_4_/AT-LX	Reduces inflammatory hyperalgesia	Formalin-/carrageenan-/CFA-induced pain	Svensson et al. (2007)
	Reverts morphine tolerance	Tail flick test	Tian et al. (2015)
	Alleviates mechanical allodynia	Neuropathic pain	Wang et al. (2014)
	Inhibits NF-kB	Hernia, DRG compressive pain, carrageenan-induced pain	Svensson et al. (2007); Sun et al. (2012); Miao et al. (2015)
	Reduces TNF-α , IL-1β , and IL-6 production	Carragenaan-treated mice	Abdelmoaty et al. (2013); Hu et al. (2012); Wang et al. (2014)
	Counteracts nociceptin action	Mouse air pouch	Serhan et al. (2001)
	Interferes with IL-1β maturation	Neuropathic pain	Li et al. (2013)
	Inhibits activation of spinal microglia	SCI rat model	Martini et al. (2016)
**DHA-derived SPMs**	**Effects**	**Disease model**	**Reference**
AT-RvD_1_	Reduces weight-bearing asymmetry and hind paw withdrawal	MIA	Huang et al. (2017)
	Abates the production of pro-inflammatory factors e.g. TNF-α and IL-1β	Arthritis	Lima-Garcia et al. (2011)
	Exerts anti-neuroinflammatory action mediated by downregulation of 5-LOX activating protein (FLAP)	Carragenaan-treated mice	Meesawatsom et al. (2016)
17(*R*)-HDHA	Is associated with pain perception	OA	Valdes et al. (2017)
	Blunts astrogliosis in the spinal cord	OA	Huang et al. (2017)
	Down regulates NF-kB and COX2 in dorsal horn of lumbar spinal cord and in the neurons of DRG	OA/sciatica	Lima-Garcia et al. (2011); Liu et al. (2016)
ALX (receptor)	Spinal overexpression	Carrageenan-induced pain	Meesawatsom et al. (2016); Huang et al. (2017)
RvD_1_	Drives PPARγ-dependent analgesic effects	DM	Saito et al. (2015)
	Improves tissue regeneration due to M2 macrophages	DM	Saito et al. (2015)
	Reduces neuroinflammation and phosphorylation of NMDA receptors in the neurons of the thoracic spinal dorsal horn	Chronic pancreatitis	Quan-Xin et al. (2012)
	Prevents heat-induced pain, inflammatory hypersensitivity and nociception-related EPSC	Sepsis	Bang et al. (2010); Park et al. (2011b)
	Reduces mechanical allodynia and heat hyperalgesia	Bone tumour	Khasabova et al. (2020)
RvD_2_	Analgesic	Post-operative neuropathic pain	Huang et al. (2011); Wang and Strichartz, (2017); Zhang et al. (2018)
	Prevents heat-induced pain behaviour, inflammatory hypersensitivity and nociception-related EPSC	Sepsis	Bang et al. (2010); Park et al. (2011b)
PD_1_	Reduces glial reaction in mouse models	Sciatic nerve transection	Xu et al. (2013)
	Inhibits TRPV1 in DRG-derived neurons	Chemically/pharmacologically-induced pain	Park et al. (2011a)
	Reduces mechanical allodynia	Sciatic nerve axotomy and spinal nerve ligation	Xu et al. (2013)
	Induces the expression of BDNF	Ocular pathology	Pham et al. (2017); Pham and Bazan, (2021)
GPR37 (receptor)	Blunts inflammation, heat hyperalgesia and mechanical allodynia	GPR37^−/−^ mice	Bang et al. (2018)
MaR_1_	Inhibits the activation of DRG neurons and abates the activation of spinal NF-kB	Carrageenan-induced and radicular pain, spinal nerve ligation	Fattori et al (2019); Gao et al. (2018); Wang et al. (2020)
	Reduces mechanical hypersensitivity due to inhibition of macrophage inflammatory chemotaxis in the DRG	Arthritic pain	Allen et al. (2020)
**EPA-derived SPMs**	**Effects**	**Disease model**	**Reference**
RvE_1_	Suppresses inflammatory spinal nociception	Formalin-/carrageenan-/CFA-induced pain	Xu et al. (2010)
	Modulates opioid signalling	CFA-induced pain	Oehler et al. (2017)
	Decreases bone cancer-associated pain	Fibrosarcoma	Khasabova et al. (2020)
	Inhibits TRPV1	Peripheral nerve injury	Xu et al. (2010); Jo et al. (2016)

### AA-Derived SPMs

Lipoxins (LXs) represent the first SPMs ever characterized, with the first reports describing their beneficial role in preventing neuropathic pain dating back to the first years of the 21^st^ century. These seminal works not only described the ability of LXA_4_, and of its aspirin-triggered epimer, to counteract the action of nociceptin and to directly reduce inflammatory hyperalgesia, but also documented for the first time the expression of FPR2/ALX on spinal astrocytes (Serhan et al., 2001; Svensson et al., 2007).

Acute pro-inflammatory cytokines represent a primum movens in driving neuropathic pain, as well as a main target on which LXs act during the resolution of inflammatory pain. Indeed, intrathecal administration of LXA_4_ or AT-15-epi-LXA_4_ results in lower production of tumor necrosis factor (TNF)-α in carrageenan-induced spinal pain and in spinal astrocyte cultures (Abdelmoaty et al., 2013), in animal models of cancer-induced pain, where also interleukin (IL)-1β is modulated (Hu et al., 2012), as well as in SCI rat models, where they inhibit activation of spinal microglia (Martini et al., 2016).

Of note, a number of mechanistic studies have also investigated the intracellular pathways that mediate the therapeutic properties of LXs, unravelling a rather convoluted signalling network: AT-LXA_4_ can hinder IL-1β maturation by directly targeting NLRP1 inflammasome, which results in reduced mechanical allodynia and thermal hyperalgesia in models of nerve ligation, as well as it reverts morphine tolerance in tail flick tests (Li et al., 2013; Tian et al., 2015). In a similar study, the same lipid alleviated mechanical allodynia caused by chronic constriction nerve injury by hindering JAK/STAT signalling, thus reducing the production of TNF-α, IL-1β, and IL-6 (Wang et al., 2014). Further studies also clarified that cytokine modulation is exerted by LXs through different molecular mechanisms, in that intrathecal or systemic administration of LXA_4_ led to: i) inhibition of NF-kB, ii) disruption of JAK/STAT signalling—with subsequent downregulation of acute innate pro-inflammatory cytokines, and upregulation of anti-inflammatory cytokines such as IL-10 and TGFβ –, iii) dephosphorylation of ERK and JNK signalling in non-compressive hernia, DRG compressive pain and carrageenan-induced models of chronic pain (Svensson et al., 2007; Sun et al., 2012; Miao et al., 2015).

Further evidence of the involvement of LX-related resolution of inflammation in neuropathic pain has been provided by a body of investigations—mostly performed during the first decade of the 2000s—reporting the antinociceptive effects of annexin 1, which was known as FPR2/ALX receptor before it was deorphanized as the LXA_4_ target (Chen et al., 2014).

### DHA-Derived SPMs

DHA-derived SPMs have been thoroughly investigated in the context of pain research during the past 20 years. Treatment with AT-RvD_1_, or its precursor 17(*R*)-HDHA, results in the enhancement of RvD_2_ production while consistently reducing weight-bearing asymmetry and hind paw withdrawal in monoiodoacetate (MIA)-treated mice (Huang et al., 2017). Moreover, it hinders complete Freund’s adjuvant (CFA)-induced arthritic pain in rats by abating the production of pro-inflammatory cytokines like TNF-α and IL-1β (Lima-Garcia et al., 2011); interestingly, in the same studies, a lack of effect of SPM treatment on bona fide joint pathological features (such as joint oedema, chondropathy or synovitis) was reported, suggesting that the action on pain is the result of a direct activity on the inflammatory cues that drive nerve-mediated hyperalgic signals. Indeed, 17 (*R*)-HDHA was shown to blunt astrogliosis in the spinal cord (Huang et al., 2017), and to down-regulate NF-kB and COX2 in the dorsal horn of lumbar spinal cord and in DRG neurons (Lima-Garcia et al., 2011; Liu et al., 2016). Of note, circulating levels of 17(*R*)-HDHA were also associated with pain perception in osteoarthritis patients (Valdes et al., 2017). In line with this, an independent study has recently demonstrated that the anti-neuroinflammatory action behind the analgesic properties of AT-RvD_1_ was mediated by downregulation of the 5-LOX activating protein (FLAP) in the spinal cord of carrageenan-treated mice (Meesawatsom et al., 2016). Interestingly, pharmacological pain mouse models also displayed spinal overexpression of ALX and ChemR23, suggesting that the pro-resolving attempts of SPMs, though ultimately insufficient to confine phlogistic processes, might be exploited to treat neuropathic pain (Meesawatsom et al., 2016; Huang et al., 2017).

Remarkably, RvD_1_ and RvD_2_ have also been recently investigated with respect to their potential as analgesic agents in neuropathic post-operative pain, and their intrathecal administration has been shown to be beneficial in post-thoracotomy, lateral paw incision, skin/muscle incision and retraction surgery (Huang et al., 2011; Wang and Strichartz, 2017; Zhang et al., 2018).

Several chronic pathologies display chronic pain as a debilitating feature that comes from peripheral neuropathy, and also SPMs have been investigated in a number of paradigms of the same disorders. For instance, RvD_1_ drives peroxisome proliferator-activated receptor-γ (PPARγ)-dependent analgesic effects in *db/db* mice, that lack the leptin receptor (Saito et al., 2015). In this animal model of diabetes the effect of RvD_1_ was also associated to improved tissue regeneration via restoration of rosiglitazone-induced differentiation of M2 macrophages, which is impaired in *db/db* mice (Saito et al., 2015). In addition, RvD_1_ counteracted chronic pancreatitis-induced mechanical allodynia in trinitrobenzene sulfonic acid-treated rats by reducing neuroinflammation and phosphorylation of N-methyl D aspartate (NMDA) receptors in neurons of the thoracic spinal dorsal horn (Quan-Xin et al., 2012). Finally, RvD_1-5_ reduced mechanical allodynia in paclitaxel-treated mice, a well-known pharmacological model of chemotherapy-induced peripheral neuropathy (Luo et al., 2019).

Interestingly, although the inhibition of the inflammatory/neuroinflammatory cues that hyperactivate nociceptive routes is considered a main mechanism mediating analgesic properties of SPMs, accumulated evidence has also demonstrated a more direct action on nervous pain transmission, as well as an interaction with other systems that control neuropathic pain.

Of note, SPMs can act on TRP channels expressed on the surface of sensory neurons, with RvD1 and in particular RvD_2_ is able to directly inhibit TRP vanilloid subtypes 1, 3, 4 (TRPV1, TRPV3, and TRPV4) and ankyrin 1 (TRPA1) channels, thus preventing *in vivo* heat-induced pain, inflammatory hypersensitivity, agonist-induced pain and nociception-related excitatory postsynaptic currents (Bang et al., 2010; Park et al., 2011b). Interestingly, also an interaction with opioid receptor has been described. Oehler and colleagues have recently reported that the antinociceptive properties of RvD_1_-and ChemR23-associated signalling can be abolished by pharmacological blockade of the μ-opioid peptide receptor (MOR) (Oehler et al., 2017). Such an effect did not seem to be related to a direct activation of MOR by SPMs, nor did SPMs induce any release of MOR ligands like β-endorphin; thus, it was suggested that a cross-talk between MOR and TRP channels signalling was taking place (Oehler et al., 2017). Furthermore, SPMs might alleviate neuropathic pain by acting synergically with other immunomodulating lipids, such as endocannabinoids, in that not only intrathecal administration of RvD_1_ led to reduced mechanical allodynia and heat hyperalgesia in bone tumour-bearing mice, but also improved the production of *N*-arachidonoylethanolamine (AEA, also known as anandamide) and 2-arachidonoylglycerol (2-AG) in the spinal cord of these animals (Khasabova et al., 2020). Of note, RvD2 receptor GPR18 is known to engage endocannabinoids, which have been postulated as possible adjuvants in pain treatment (Guerrero-Alba et al., 2019).

Also (neuro)protectins (PDs) have been studied in the past decade with respect to their ability to blunt neuropathic pain. Interestingly, GPR37 has only recently been characterized as receptor of PD_1_ (Bang et al., 2018), but it was known for its antinociceptive properties ways before the discovery of its pro-resolving ligand. GPR37 is expressed in macrophages, suggesting that these immune cells play a role in modulating neuroinflammatory pain, as also demonstrated by the presence of GPR37-positive macrophages in mouse samples of spinal cord (Bang et al., 2018, 018). Furthermore, local inflammation upregulates GPR37 in macrophages, and ablation of GPR37 in mice leads to delayed resolution of inflammatory pain (Bang et al., 2018). Interestingly, microglial cells do not express GPR37, suggesting that this receptor might exert its analgesic effects through the secretion of some macrophage-related soluble factors (e.g., IL-10), which in turn blunt peripheral neuroinflammation and reduce nociception in pain-sensing primary sensory neurons (Bang et al., 2018; Chen et al., 2020). Furthermore, previous studies reported a reduction of glial reaction upon local injection of PD_1_ in mouse models of sciatic nerve transection, suggesting that the latter SPM might act through multiple pathways, yet to be fully understood (Xu et al., 2013). Consistently, GPR37^−/−^ mice display enhanced mechanical and cold allodynia, as well as thermal hyperalgesia during bacterial infections, while macrophages primed with GPR37 agonists reduce mechanical allodynia in the same GPR37^−/−^ mice (Bang et al., 2021). Also PD_1_ has been demonstrated to counteract neuropathic pain by directly targeting sensory nociception via TRPV1 inhibition in DRG neurons (Park et al., 2011a), and by reversing nerve injury-induced spinal cord synaptic plasticity in nerve trauma (Xu et al., 2013). Moreover, PD_1_ is able to induce the expression of brain-derived neurotrophic factor (BDNF) in injured cornea, where it might prevent neuropathic pain by preventing corneal nerves damage (Pham et al., 2017; Pham and Bazan, 2021). Recently, the PD_1_ synthetic analog 3-oxo-PD_1n-3 DPA_ has been reported to exert antinociceptive activity on neuropathic pain associated to streptozocin-induced diabetes in mice (Irina Nesman et al., 2021).

To date, only a few studies have interrogated possible antinociceptive actions of MaRs in models of neuropathic pain. Intrathecal administration of MaR_1_ inhibits the activation of DRG neurons and abates activation of spinal NF-kB and production of TNF-α and IL-1β, resulting in reduced mechanical and thermal hyperalgesia in CFA- and carrageenan-induced pain paradigms, up to 3–5 days after the treatment (Fattori et al., 2019) and in spinal nerve ligation models (Gao et al., 2018). MaR_1_ also reduces mechanical allodynia in rat models of radicular pain (Wang et al., 2020), as well as chemotaxis of inflammatory cells in the calcitonin gene-related peptide (CGRP)-releasing DRG neurons (Fattori et al., 2019). In line with this, a recent investigation has shown that intraperitoneal administration of MaR_1_ in a K/BxN transfert-based model of arthritic pain led to reduced mechanical hypersensitivity, due to inhibition of macrophage inflammatory chemotaxis in the DRG (Allen et al., 2020). Similarly to other SPMs, therapeutic properties of MaR_1_ have been linked to its ability to act on vanilloid receptors. Indeed, this lipid showed anti-nociceptive properties in capsaicin-induced spinal and cranial pain, by specifically acting on TRPV1- (but not TRPA1-) induced currents (Serhan et al., 2012; Park, 2015). It seems noteworthy that MaR1 is coupled to G_αI_ proteins, in that pertussis toxin (PTX) reverses its therapeutic activity (Serhan et al., 2012; Park, 2015). This observation has been confirmed when LGR6 was recognized as the G protein-coupled receptor responsible for the effects of MaR_1_ (Chiang et al., 2019). It should be stressed that LGR6 has not yet been investigated in neuropathic pain, where it might represent a valuable target for future therapeutic strategies.

### EPA-Derived SPMs

E-series resolvins share a number of anti-nociceptive properties with DHA-derived SPMs. RvE_1_ was firstly described to suppress inflammatory spinal nociception in CFA-, carrageenan- and formalin-induced pain (Xu et al., 2010). In these paradigms, pain manifests as a biphasic process, the first phase coming from activation of nociceptive receptors, and the second manifesting as a change in the activity of the spinal neurons following the first phase (Ji et al., 1999). Preemptive administration of RvE_1_ in formalin-induced pain was only effective in the second phase of pain, suggesting an action of this SPM on central nociceptive signals (Xu et al., 2010). Furthermore, RvEs are also able to modulate pain by interacting with other systems involved in nociceptive signalling. For instance, co-expression of ChemR23 and TRPV1 in DRG neurons has been described, suggesting that inhibition of TRPV1 might depend on ChemR23 stimulation by RvE_1_ (Xu et al., 2010; Jo et al., 2016). Should this interaction be confirmed, SPM receptors and TRP channels might be part of an homeostatic system involved in the genesis of inflammatory neuropathic pain on nociceptive and DRG neurons. Furthermore, RvEs share also with DHA-derived SPMs the ability to modulate signalling of opioids (Oehler et al., 2017), as well as of other lipids, as supported by the observation that RvE_1_ (like RvD_1_) induces spinal synthesis of endocannabinoids, and together they abate bone cancer-associated pain (Khasabova et al., 2020).

The potential role of ChemR23-dependent signalling in antinociception is also advocated by the finding that chemerin (its firstly identified ligand), or peptide derivatives of this molecule, display analgesic properties by reducing spinal pain (Xu et al., 2010; Doyle et al., 2014), and inhibiting activation of C-fibers (Dickie and Torsney, 2014); Interestingly, RvE_1_ is the only SPM as yet known that is able to promote resolution not only by engaging its own receptor—which in turn contributes to the deactivation of the inflammatory wave—but also by acting as a direct antagonist of the LTB_4_ receptor BLT1. The latter represents a prominent member among pro-inflammatory eicosanoids that participate to inflammation and pain (Inoue and Tsuda, 2018), indicating that resolution of neuropathic pain might indeed be a convoluted process, where SPMs act in synergy, directly or indirectly, with other antinociceptive signals.

## Conclusion

Neuropathic pain represents a common and invalidating impairment that undermines the overall quality of life of many patients, for which there is an unmet need of therapeutic treatments especially in the long term. Chronic inflammation represent a very common cause of neuropathic pain, and SPMs are powerful lipid signals that control the resolution of inflammation and prevent many aberrancies of uncontrolled, or prolonged, phlogistic processes including pain. Anti-nociception is exerted by SPMs via three main mechanisms by: i) directly controlling activation of immune cells, their chemotaxis and the release of cytokines; ii) targeting the activation of TRP channels (mostly TRPV1 and TRPA1), for which SPMs represent the first endogenous inhibitors ever described (Park et al., 2011b, 1; Lim et al., 2015); and iii) interacting with opioid signalling.

These observations suggest that SPMs exert a rather pleiotropic activity on the onset of pain, which is achieved by targeting the neuroinflammatory cues that lead to hypersensitivity of nociceptive fibers, and by directly affecting the neuronal processes that lead to pain signal release. Of note, different SPMs seem to drive resolution of inflammatory pain by modulating distinct targets (Fonseca et al., 2017; Valdes et al., 2017; Luo et al., 2019), overall suggesting that these lipids might intervene in different moments, contexts, and possibly types of injuries in order to alleviate pain. One should also consider that, despite the large number of SPMs that have been identified so far, only a handful of SPM receptors have been characterized, and some of them have never been investigated in pain paradigms: one such example is GPR101, which engages the n-3 docosapentaenoic acid (DPA)-derived analogue of RvD5 (Flak et al., 2020). Furthermore, several SPM receptors which have not yet been investigated in pain (see Figure 1C) are nonetheless expressed in neurons, astrocytes and microglia (Tiberi and Chiurchiù, 2021), and might well play a role in the modulation of neuropathic pain.

Oxidative stress is another mechanism that drives inflammation and neuroinflammation, as well as neuropathic pain. Of note, a growing body of evidence has demonstrated the ability of SPMs to blunt the damage caused by excessive production of reactive oxygen and nitrogen species (ROS and RNS, respectively), either by reducing their production or by potentiating anti-oxidative defence systems such as the Kelch-like ECH associated protein 1 (Keap1)/nuclear factor erythroid 2-related factor 2 (Nrf2) pathway, superoxide dismutase (SOD) and glutathione peroxidase (GSH-PX) (Leuti et al., 2019). Of note, aberrant production of ROS and RNS, coming from deviant and unresolved inflammatory response, not only directly damages sensitive structure that contribute to the transmission of the nociceptive signals (e.g., DRG neurons) but also perpetuates a vicious cycle by activating glial cells, inducing leukocyte recruitment and overall fostering the inflammatory torrent that ultimately leads to neuropathic inflammatory pain. As a result, unbalanced oxidative stress plays a major role in peripheral neuropathies such as those caused by diabetes, alcohol abuse or chemotherapy (Carrasco et al., 2018). Interestingly, TRP channels can be activated by free radicals (Carrasco et al., 2018), suggesting not only that they represent a pivotal element in the genesis of neuropathic pain, but also that SPMs might abate pain by acting on these channels both directly (as described in the present review) and indirectly by interfering with ROS/RNS-mediated damage. To date, however, the direct relationship between pro-resolving lipids, oxidative stress and neuropathic pain has never been directly addressed as a whole, and though strongly suggested by accumulated evidence, it remains a merely speculative mechanism.

It should be noted that microglial cells play a crucial role in mediating spinal pain in several pathologies, and SPMs have consistently been reported to act on the same cells by multiple mechanisms (Tiberi and Chiurchiù, 2021).

Also of relevance, not only SPMs represent very well-tolerated molecules, but some studies also reported their analgesic effect at much lower doses than morphine or COX2 inhibitors (Xu et al., 2010).

It should be noted that SPMs-related dysfunctions have been described in a number of pathologies that feature chronic neuropathic pain as the main or a collateral feature, such as SCI, Parkinson’s disease and MS (Prüss et al., 2013; Bisicchia et al., 2018; Krashia et al., 2019; Kooij et al., 2020; Derada Troletti et al., 2021), suggesting that these lipids represent valuable targets for both a better understanding of the pathogenesis of neuropathic pain and the future development of therapeutic strategies that counteract hyperalgic signals by enhancing resolution of inflammation. On a final note, most of the investigations published so far were performed by administered SPMs topically, intraperitoneally or intrathecally: additional studies that investigate the efficacy of these lipids through other administration pathways (e.g., oral, sublingual) will be required in order to better evaluate their antinociceptive properties, as well as to formulate new therapeutic strategies; on the other hand, DHA and EPA, as well as SPM precursors are currently commercialized as integrators or nutraceuticals, and a number of studies have reported the beneficial effects of PUFA administration in pain animal models as well as in patients (Ko et al., 2010; Galán-Arriero et al., 2017; Silva et al., 2017).
